# Two Outbreaks of Invasive Pneumococcal Disease in Nursing Homes in Gipuzkoa, Northern Spain

**DOI:** 10.3390/vaccines13060570

**Published:** 2025-05-26

**Authors:** José María Marimón, Ayla Manzanal, Olatz Mokoroa, Lorea Alvarez, Maite Rekalde, Diego Vicente

**Affiliations:** 1 Biogipuzkoa Health Research Institute, Infectious Diseases Area, Microbiology Department, Donostia University Hospital, Donostialdea Integrated Health Organization, 20014 Donostia-San Sebastian, Spain; 2 Department of Preventive Medicine, Faculty of Medicine, University of the Basque Country (UPV/EHU), 20014 Donostia-San Sebastian, Spain; 3 Ministry of Health of the Basque Government, Sub-Directorate for Public Health and Addictions of Gipuzkoa, 20013 Donostia-San Sebastian, Spain; 4 Biogipuzkoa Health Research Institute, Epidemiology of Chronic and Communicable Diseases Group, 20014 Donostia-San Sebastian, Spain; 5 Biogipuzkoa Health Research Institute, Infectious Diseases Area, Infectious Epidemiology and Antimicrobial Resistance Group, 20014 Donostia-San Sebastian, Spain; maiteelisa.rekaldehernando@bio-gipuzkoa.eus

**Keywords:** *Streptococcus pneumoniae*, outbreak, invasive pneumococcal disease, pneumococcal polysaccharide vaccine 23

## Abstract

**Background**: The aging of the population has increased the number of frail people living in long-term care facilities, underscoring the need for continuous updates in infectious diseases prevention strategies. The aim of this study was to analyze two pneumococcal disease outbreaks in elderly residences in Gipuzkoa, northern Spain, their impact on residents, and the containment measures implemented. **Material and methods**: The outbreaks took place in 2023 and in 2024 in two residences of 111 and of 155 residents, respectively. Diagnosis was based on clinical criteria, radiological findings, and microbiological techniques. Pneumococcal isolates were characterized by whole-genome sequencing. **Results**: The outbreaks involved five and six residents, respectively. Most residents in both facilities had been vaccinated with the pneumococcal polysaccharide 23-valent vaccine (PPV23) more than five years prior. The median attack rates were 4.5% and 3.9%, lower than those reported in similar outbreaks. The adopted infection transmission prevention measures successfully limited the spread of the outbreaks. **Conclusions**: PPV23 vaccination did not prevent invasive pneumococcal infection in the affected residents. The vaccination of elderly people living in long-term care facilities with 20-valent and 21-valent pneumococcal conjugated vaccines should be evaluated as a new preventive measure.

## 1. Introduction

*Streptococcus pneumoniae* is a Gram-positive diplococcus that forms part of the human respiratory microbiota but can also be a pathogenic bacterium causing a wide range of diseases, among which meningitis and sepsis are the most severe forms. The use of pneumococcal conjugate vaccines (PCV) since 2000 for routine childhood vaccination has changed the epidemiology of pneumococcal infections in countries where it has been used, with a decrease in infections caused by vaccine serotypes and increases in the incidence of non-vaccine serotypes [1]. In Gipuzkoa, PCV vaccines for childhood vaccination have been available since 2001; 13-valent PCV (PCV13) was introduced in the childhood vaccination schedule in 2015 (Supplementary Materials, Figure S1). For adult vaccination, pneumococcal polysaccharide vaccine 23 valent (PPV23) was introduced in routine vaccinations at 65 years of age in 2007. However, although vaccination coverage of children exceeds 95%, coverage in elderly populations has not reached 25%. This figure is similar to that reported in other recent Spanish studies, in which the pneumococcal vaccination rate in people over the age of 65 was 29%, much lower than the 80% rate for influenza vaccination [2].

*S. pneumoniae* is most commonly transmitted by direct contact with the respiratory secretions of a carrier or infected person; nevertheless, the number of pneumococcal outbreaks reported is not as high as that of other respiratory transmitted pathogens [3]. Two types of pneumococcal outbreaks are commonly described: those that affect a large number of people, lasting over time and in large geographic regions like the one caused by serotype 4 ST801 that especially affected shipyard workers from Finland, Norway, and Northern Ireland [4]; or the one caused by serotype 2 in Israel, which lasted for more than 10 years [5]. The other types of outbreaks described are those limited to small closed environments, like prisons [6], nursing homes [7,8] or hospital wards [9], which affect fewer people in shorter periods of time. In a review on pneumococcal outbreaks, Zivich et al. reported a median attack rate of 7% (IQR 2.4–13%), a median case-fatality ratio of 12.9% (IQR 0–29.2%), with older adults being the age group most affected with different models of transmission, including carriers, infected individuals, and medical devices [10]. Among the prevention measures, pneumococcal vaccination, antibiotic prophylaxis, and infection control measures have been adopted in outbreaks in close settings [11,12]. Vaccination or revaccination is likely the best measure with which to prevent the initial occurrence of outbreaks [10].

In this work, we describe two outbreaks of invasive pneumococcal disease (IPD) in two different nursing homes in Gipuzkoa, northern Spain that occurred in 2023 and 2024, focusing on patients’ outcomes, vaccination status, and the measures adopted to contain the outbreaks.

## 2. Materials and Methods

### 2.1. Outbreaks Description

In this study, “confirmed cases” were defined as patients residing in the same nursing home in the period when the outbreak took place, having respiratory symptoms compatible with lower respiratory tract infection or IPD (fever, chills, cough, dyspnea, fatigue); a Chest X-ray compatible with pneumonia (consolidation, pleural effusion); and microbiological confirmation such as a positive culture (blood, sputum) or a positive pneumococcal urine antigen test (UAT). “Probable cases” were defined as patients with a clinical diagnosis of pneumonia but without radiological or microbiological confirmation.

### 2.2. Microbiological Procedures

Blood cultures were performed using an automated blood culture system (BD BACTEC™ FX system; BD, Franklin Lakes, NJ, USA). Alpha-hemolytic streptococci grown on subcultured blood agar plates (Columbia blood agar COS, bioMérieux, Marcy l’Étoile, France) were identified as *S. pneumoniae* by the optochin susceptibility test. Serotyping of isolates was carried out using the SPneumostrip reverse-hybridization system (Operon SL, Zaragoza, Spain) [13]. Genotyping was performed by WGS. Antimicrobial susceptibility testing was performed by the broth microdilution method according to EUCAST guidelines [14].

For the detection of pneumococcal serotypes on carriers in the second nursing home outbreak, throat swabs were plated on Columbia blood agar and Columbia CNA agar (with nalidixic acid and colistin) enriched with 5% sheep’s blood (Becton Dickinson, Cockeysville, MD, USA), incubated overnight at 35 °C in a 5% CO_2_-enriched atmosphere. A loopful of mixed bacterial growth was resuspended in 1 mL of TE buffer (Tris 10 mM, EDTA 1 mM, pH 7.8); DNA was purified using bioMérieux’s EMAG nucleic acid extraction system. Serotypes were detected using the SPneumostrip. Samples positive for *S. pneumoniae* by the PCR were cultured again; *S. pneumoniae* strains were isolated for further studies.

### 2.3. Whole-Genome Sequencing (WGS)

A single pneumococcal colony was subcultured on blood agar plates (COS, bioMérieux) and incubated for 24 h at 35 °C in a 5% CO_2_-enriched atmosphere. Nucleic acids were extracted using Qiagen columns (QIAamp DNA blood kit, QIAGEN, Hilden, Germany). DNA was quantified using Qubit (Thermofisher Scientific, Waltham, MA, USA) and adjusted to 6 ng/mL for preparing libraries using the Illumina DNA Prep kit (Illumina, San Diego, CA, USA). Sequencing was performed on an iSeq platform. Assemblies were conducted using metSpades (Version 0.5.0) and annotated using prokka (Version 1.14.6) using the software tools available at the European Galaxy server (https://usegalaxy.eu/, accessed on 18 February 2025). Sequence types (ST) and core-genome ST (cgST) were identified by querying assembled genomes on the MLST web page (https://pubmlst.org/organisms/streptococcus-pneumoniae, accessed on 13 January 2025). The assembled genomes were deposited in Genbank as BioProject ID PRJNA1209867.

### 2.4. Ethics Statement

The study was approved by the local Ethics Committee from Gipuzkoa, reference MAR-NEU-2025-01.

## 3. Results

### 3.1. First Outbreak

The first outbreak took place in October 2023 in a nursing home in village 1 that admitted 111 residents and had 126 professionals. In total, five patients were affected: three confirmed and two probable cases (attack rates 4.5%) (Table 1). All the patients had the onset of symptoms between 17 and 21 of October 2023.

In October 2023, three consecutive cases of IPD raised the suspicions of a possible outbreak. The first case was a 94-year-old man admitted to the hospital on the morning of 17 October with dyspnea and right basal pneumonia. Due to the general deterioration of the patient, sedation was started; the patient died on the same evening. Blood cultures and UAT were positive for *S. pneumoniae.* The patient had been vaccinated with one dose of PPV23 in June 2015.

The second confirmed case was an 89-year-old man, who was admitted to hospital the afternoon of October the 17th with respiratory insufficiency and bilateral condensations and pinched costophrenic sinuses on the chest radiography (CR). The next day, blood cultures drawn in the emergency room flagged positive for *S. pneumoniae*; the UAT was also positive for *S. pneumoniae*. Antibiotic therapy, empirically started at admission with ceftriaxone and azithromycin, was maintained with only ceftriaxone 24 h later until the patient was discharged due to a good evolution on October the 25th. The patient had been vaccinated with one dose of PPV23 in October 2007.

A third patient, an 84-year-old man from the same nursing home, was admitted to the emergency room on the evening of October the 17th with dyspnea, O2 desaturation (90%), fever (37.3 °C), and a deterioration of the level of consciousness. Due to the general situation, the patient was sedated and finally died 24 h later. Blood cultures drawn at admission were negative and no urine samples were collected. The patient had been vaccinated with one dose of PPV23 in July 2007.

A fourth patient, a 92-year-old woman, was admitted to hospital on the evening of October the 19th, with basal pneumonia with pleural effusion. Blood cultures and pneumococcal UAT collected at admission were negative. On the sputum, a rhinovirus was detected. After 4 days of antibiotic treatment with amoxicillin–clavulanate 500 mg/125 mg (AMC), the patient was discharged to the nursing home. The patient declined vaccination when it was offered in November 2015.

The last confirmed case was a 91-year-old man admitted to the hospital the morning of October the 21st with dyspnea and a left basal infiltrate on the CR. Blood cultures were negative; however, pneumococcal UAT at admission was positive. On the high-quality sputum collected on the morning of the 22nd (more than 25 leukocytes and less than 10 epithelial cells per low powered field), abundant growth of *S. pneumoniae* together with *Haemophilus influenzae* was observed. Antibiotic therapy was started with ceftriaxone and azithromycin and changed to levofloxacin at discharge 3 days later. The patient had been vaccinated with one dose of PPV23 in September 2007.

The three *S. pneumoniae* isolates from patients from the nursing home (the blood cultures of patient 1 and 2, and the sputum of patient 5) were serotype 31, ST1766, and were susceptible to all the antibiotics tested (penicillin, erythromycin, levofloxacin, vancomycin, linezolid). The first two isolates were cgST 71011; however, the third one, isolated from the sputum, was cgST 59896 (Table 2). A serotype 31 isolate from a non-related patient isolated in 2022 was used as a control for WGS and was ST1684 and cg 40123.

#### Outbreak Measures Adopted in the Nursing-Home

On 23 October, measures were established in the nursing home to control the outbreak based on the guidelines provided by the Sub-Directorate of Public Health and Addictions of Gipuzkoa. These measures included the mandatory use of masks by staff to minimize the risk of transmission. Hygiene protocols were reinforced, with an emphasis on proper respiratory etiquette, such as covering the mouth and nose when coughing or sneezing, and by promoting frequent and thorough hand hygiene using soap and water or alcohol-based hand sanitizers. These measures were maintained for 2 months.

Additionally, healthcare professionals were advised to closely monitor residents for early signs of respiratory illness. For those presenting symptoms suggestive of a respiratory infection, the prompt initiation of treatment with amoxicillin was recommended to ensure effective management and prevent complications. These actions aimed to protect the health of the most vulnerable individuals and to limit the spread of the infection within the facility.

No new cases were detected.

### 3.2. Second Outbreak

The second outbreak took place in a nursing home that accommodates 155 residents and 120 staff personnel for residential care and another 60 for daily care in village 2. The outbreak comprised two confirmed and two probable cases in April and another two confirmed cases of IPD in June (attack rate 3.9%). In April, two residents were found to be pharyngeal carriers of the pneumococcus responsible for the outbreak in an epidemiological survey who were not considered as cases.

The first case was diagnosed on 30 March 2024. After 4–5 days of malaise and dyspnea, the patient, an 88-year-old man, was transferred from the nursing home to the hospital emergency room with fever and hypoxemia (O2 saturation 92%). CR showed a large condensation in the left upper lobe. Blood cultures on admission were positive for *S. pneumoniae* serotype 8. Pneumococcal UAT was also positive. The patient was treated with ceftriaxone 2 g/24 h and levofloxacin 500 mg/24 h for seven days, when he was discharged due to good progress. Antibiotic treatment with levofloxacin was continued for a further 5 days. The patient had been correctly vaccinated with one dose of PPV23 in September 2007.

The second case, an 80-year-old male, was admitted to the emergency department on the morning of 6 April 2024 from the nursing home due to dyspnea at rest of a few hours’ duration, fever (37.2 °C) and hypoxia (O2 saturation of 92%). The CR showed a right basal condensation and the UAT was positive for *S. pneumoniae*; thus, antibiotic treatment with ceftriaxone and azithromycin was started. Due to the poor clinical evolution, the patient was sedated that same night and finally died on 8 April. The blood cultures collected in the emergency department were also positive for *S. pneumoniae* serotype 8. The patient had no records of pneumococcal vaccination.

Another two cases of respiratory infections on the same floor were detected that were empirically treated with amoxicillin; however, no microbiological cultures or UAT were performed. These two cases were considered as “probable cases”.

#### Outbreak Measures Adopted in the Second Nursing-Home

On 11 April, the Sub-Directorate of Public Health and Addictions of Gipuzkoa implemented the same measures as in the previous outbreak: reinforcement of hygiene protocols and mandatory use of face masks for all staff members. Furthermore, in response to an outbreak caused by *S. pneumoniae* serotype 8, vaccination with PPV23 was recommended for all unvaccinated residents in the nursing home. In addition, on the floor affected by the outbreak, an additional dose of PPV23 was recommended for those residents who had received a dose more than five years ago. One resident vaccinated in 2009 rejected the additional dose and another two residents refused any vaccination with PPV23.

Reinforced hygiene measures and the use of masks continued until two weeks after all residents had received the corresponding vaccine dose (first week of May).

Since the four patients lived on the same floor of the residence, a control of pneumococcal carriers was carried out on a voluntary basis on half of the residents of that floor. Many residents were reluctant to collect the pharyngeal sample due to previous experiences with the COVID-19 pandemic. In total, pharyngeal samples were taken from 22 residents, in whom the presence of pneumococcus was detected by PCR in 5, two of which were serotype 8. Both isolates could be recovered from the blood agar plate; WGS was performed. The two residents were asymptomatic and did not receive any antibiotic treatment.

Another two cases of invasive disease caused by serotype 8 isolates occurred in the same residence in June 2024. These two patients were admitted to the nursing home after the lifting of the control measures imposed between April and May and were roommates.

On 7 June, a 92-year-old woman was referred to the emergency departments of the hospital with moderate-severe cognitive impairment with respiratory insufficiency in the context of sepsis of respiratory origin. The CR revealed condensation in the right lobe. The UAT was positive for *S. pneumoniae*; an *S. pneumoniae* serotype 8 grew in the blood culture drawn at admission. The patient was treated with ceftriaxone 1 g/24 h for seven days, when she was discharged to the nursing-home with cefditoren. The patient had been correctly vaccinated with the PPV23 in 2007.

On 13 June, an 83-year-old woman, with deterioration of her general condition and clinical symptoms of respiratory infection with hypoxia was referred from the nursing home to the hospital for admission. The CR revealed a right pleural effusion. Blood cultures were not performed; the routine sputum culture yielded scant growth of *S. pneumoniae* serotype 8. The patient had been positive for SARS-CoV-2 infection two days before. Diagnosed with COVID and bacterial superinfection, the patient was admitted for 7 days and treated with ceftriaxone until being discharged to the residence. There was no record of pneumococcal vaccination for this patient.

After these two new cases, hygiene measures were reinforced; active surveillance was initiated for a month. No new cases were detected.

The four isolates from the confirmed cases and the two isolated from the carriers were serotype 8, ST1629 and had the same cgST 61033 by WGS. An *S. pneumoniae* serotype 8 isolated in 2020 and used as a control of the WGS was ST53 and cg59927.

## 4. Discussion

In this work, we report two pneumococcal outbreaks in nursing homes after the SARS-CoV-2 pandemic. The description of pneumococcal outbreaks in nursing homes are rarely documented in the literature, with most reports focusing on hospitals, likely due to observational biases [10].

The two outbreaks involved two different pneumococcal serotypes: serotype 8, which is included in the PPV23, and serotype 31, which is not. Contrary to other pneumococcal outbreaks [15], most residents of both nursing homes had been vaccinated several years previously.

The first outbreak caused by *S. pneumoniae* serotype 31 ST 1766 affected five residents. Although a respiratory isolate showed minor genetic differences compared to invasive isolates, it was considered part of the outbreak based on its epidemiological link and because it had the same serotype and ST. An increase in the prevalence of serotype 31 ST1766 in nasopharyngeal carriers after the introduction of PCV13 has been recently described in Spain [16]. In a study conducted in Madrid between 2007 and 2020, serotype 31 was the serotype with the highest mortality rate at 29.5% [17]. In our study, two of the residents infected with this serotype died during the outbreak.

The second outbreak was caused by a serotype 8 ST1629 strain, a rare genotype within this serotype previously recorded only once in the MLST database (accessed 24 March 2025) in a 96-year-old woman in the UK in 2005. Remarkably, the strain causing this outbreak persisted in the residence for at least two months, causing a two-wave outbreak, one in April and the other in June; this was confirmed by WGS, who characterized all the isolates as cgST61033. Additionally, the outbreak strain was identified in asymptomatic carriers, emphasizing the potential of conjugate vaccines to limit transmission in close-contact settings.

Serotype 8 is included in PPV23; however, the vaccination did not protect residents with the infection despite the correct use of the vaccination schedule. In addition, serotype 8 is currently the most frequent serotype causing IPD in adults in Spain, accounting for nearly 20% of IPD cases [17,18]. The high incidence of serotype 8 pneumococcal disease in our region and the lack of protection for residents against serotype 8 IPD, as a consequence of the waning of protective antibodies in elderly people years after vaccination [19,20], were probably the cause of this outbreak. A serotype 8 outbreak in a residential care home in England in 2012 demonstrated a high attack rate (65%) and resulted in two deaths among 15 infected residents [19]. In that outbreak, as in ours, all residents had been vaccinated years previously with PPV23, which seemed insufficient for preventing the infections and transmission of the strain responsible for the outbreak.

The attack rates (4.5% and 3.9%) were lower than the median of 7% described in a review of 86 respiratory *S. pneumoniae* outbreaks [10]. The rapid establishment of preventive measures (use of masks and enhanced hygiene measures), which did not include antibiotic prophylaxis or revaccination of residents, seemed to be sufficient to prevent the spread of the outbreak strains. However, two new cases appeared in the second outbreak after prevention measures were lifted, potentially attributable to staff carriage or environmental contamination. Since respiratory infections can spread easily in closed settings, rapid implementation of infection control and other preventive measures are of the utmost importance, especially in settings with vulnerable populations such as the elderly [11]. The role of antibiotic chemoprophylaxis is more controversial, with some guidelines in favor of this [12]; however, in the two outbreaks described here, as in others [8], infection control measures were sufficient to control the outbreaks. The case fatality ratio (2/5 and 1/6) were higher than the median of 12.9% described in another study [10], likely reflecting the advanced age and comorbidities of the affected residents. The three deceased patients did not receive antibiotic treatment, as a joint decision was made by their relatives and the medical team not to perform further therapeutic interventions, given the patients’ advanced age and the significant deterioration of their health.

As a T-cell-independent polysaccharide vaccine, PPV23 does not induce a booster response; the efficacy of revaccination in older adults has not been demonstrated [21]. PPV23 provides protection that generally wanes after 5 years [20], while PCVs provide longer-lasting (probably more than 15 years in adults) and more effective protection against the serotypes included in them [22,23]. Most of the patients with IPD described in our work, including those who died, had been vaccinated several years previously with the PPV23. In Spain, PPV23 is recommended for people over 2 years of age; revaccination is only recommended for people with risk conditions for IPD. Currently, the ACIP recommends the use of either PCV20 or PCV21 alone, or PCV15 in series with PPV23, for all adults aged ≥50 years [24,25]. Today, serotype 31 is only included in the PCV21.

## 5. Conclusions

The report of these two recent outbreaks in two residences with high PPV23 vaccination rates highlight the importance of vaccination strategies in the era of high-valent pneumococcal conjugate vaccines. The waning immunity of PPV23 with time makes better protection for the elderly necessary, especially for those living in long-term care facilities, as they are at higher risk for IPD and death than elderly people living in the community [26]. The high serotype coverage, together with the possibility of limiting transmission, make PCVs a promising alternative for these new preventive strategies.

## Figures and Tables

**Table 1:** Demographic, clinical, and microbiological data of patients with *S. pneumoniae* infections associated with two outbreaks in Gipuzkoa, northern Spain.

Outbreak-Patient	Age/Gender	Admission Date	Admission Reason	Chest X-Ray	Biomarkers ^a^	Virus ^b^	pUAT ^c^	Blood Culture	Sputum Culture	Pharyngeal Culture	Outcome	Antibiotic Treatment
	*S. pneumoniae* serotype 31 outbreak											
1-1	94, male	17 October 2023	Dyspnea	Consolidation of the right lower and middle lobes	CRP: 140 PCT: 10	negative	positive	*S. pneumoniae*	ND ^d^	ND	Exitus	
1-2	89, male	17 October 2023	Respiratory infection	Bilateral consolidation and costophrenic angle blunting	CRP: 163.5 PCT: 4.95	negative	positive	*S. pneumoniae*	ND	ND	Discharged (25 October 2023)	ceftriaxone
1-3	84, male	17 October 2023	Dyspnea, septic shock	ND	ND	negative	ND	negative	ND	ND	Exitus	
1-4	92, female	19 October 2023	Increased respiratory secretions, dyspnea	Left basal consolidation	ND	rhinovirus	negative	negative	ND	ND	Discharged (23 October 2023)	amoxicillin-clavulanate
1-5	91, male	21 October 2023	Increased respiratory Secretions, dyspnea	Left lower-lobe consolidation	CRP: 50	negative	positive	ND	*S. pneumoniae H. influenzae*	ND	Discharged (24 October 2023)	ceftriaxone + azithromycin
	*S. pneumoniae* serotype 8 outbreak											
2-1	88, male	30 March 2024	Malaise, fever, low O2 saturation	Left upper-lobe consolidation	CRP: 275 PCT: 5.85	negative	positive	*S. pneumoniae*	ND	ND	Discharged (5 April 2024)	ceftriaxone + levofloxacin
2-2	80, male	6 April 2024	Sudden dyspnea at rest	ND	CRP: 432 PCT: 4.1	negative	positive	*S. pneumoniae*	ND	ND	Exitus	
2-3 (carrier 1)	69, male	17 April 2024	Pharyngeal carrier	-	-	-	-	-	-	*S. pneumoniae*		
2-4 (carrier 2)	89, female	17 April 2024	Pharyngeal carrier	-	-	-	-	-	-	*S. pneumoniae*		
2-5	92, female	7 June 2024	Moderate/severe cognitive impairment	Right lower-lobe consolidation	CRP: 235 PCT: 58.8	negative	positive	*S. pneumoniae*		ND	Discharged (14 June 2024)	ceftriaxone + azithromycin
2-6	83, female	13 June 2024	Dyspnea and COVID	Right pleural effusion	CRP: 110	SARS-CoV-2	ND	ND	*S. pneumoniae*	ND	Discharged (21 June 2024)	ceftriaxone

^a^ CRP: C-reactive protein (mg/dL). normal value: <1.0 mg/dL. PCT: procalcitonin (ng/mL). Normal value: 0.5 ng/mL. ^b^ Respiratory virus detection on pharyngeal sample (influenza virus, parainfluenza virus, respiratory syncytial virus, adenovirus, human metapneumovirus, rhinovirus, SARS-CoV-2). ^c^ pUAT: Pneumococcal urine antigen test. ^d^ ND: Not done.

**Table 2:** Serotypes, sequence-types (ST) and core-genome ST (cgST) of the pneumococcal isolates causing the two outbreaks in nursing homes in Gipuzkoa, northern Spain.

Outbreak-Patient	Date	Serotype	MLST	cg Closest Profile	Loci Matched
1-1	17 October 2023	31	ST1766	cgST 71011	1192/1222 (97.5%)
1-2	17 October 2023	31	ST1766	cgST 71011	1192/1222 (97.5%)
1-5	21 October 2023	31	ST1766	cgST 59896	1192/1222 (97.5%)
control	19 February 2022	31	ST1684	cgST 40123	1149/1222 (94.0%)
2-1	30 March2024	8	ST1629	cgST 61033	1207/1222 (98.8%)
2-2	06 April 2024	8	ST1629	cgST 61033	1208/1222 (98.9%)
2-3 (carrier 1)	17 April 2024	8	ST1629	cgST 61033	1207/1222 (98.8%)
2-4 (carrier 2)	17 April 2024	8	ST1629	cgST 61033	1208/1222 (98.9%)
2-5	07 June 2024	8	ST1629	cgST 61033	1208/1222 (98.9%)
2-6	12 June2024	8	ST1629	cgST 61033	1207/1222 (98.8%)
control	17 January 2020	8	ST 53	cgST 59927	1215/1222 (99.4%)

## Data Availability

Assembled genomes are deposited in Genbank as BioProject ID PRJNA1209867.

## Supplementary Materials

The following supporting information can be downloaded at: https://www.mdpi.com/article/10.3390/vaccines13060570/s1, Figure S1: Serotypes inlcuded in the different valent (indicated by the number) pneumococcal conjugate vaccines (PCV) and polysaccharide vaccine (PPV).
