# Supplementary material for: Two Outbreaks of Invasive Pneumococcal Disease in Nursing Homes in Gipuzkoa, Northern Spain
Source: Vaccines (Basel). 2025 May 26;13(6):570. doi: 10.3390/vaccines13060570 (PMC12197688; doi:10.3390/vaccines13060570)
Supplement: Supplementary file 1 [file vaccines-13-00570-s001.zip › vaccines-3619788-supplementary.pdf]

Figure S1. Serotypes inlcuded in the different valent (indicated by the number) pneumococcal conjugate vaccines (PCV) and polysaccharide vaccine (PPV).

Serotype	PCV7	PCV10	PCV13	PCV15	PCV20	PPV23	PCV21	Serotype
1		X	X	X	X	X		1
2						X		2
3			X	X	X	X	X	3
4	X	X	X	X	X	X		4
5		X	X	X	X	X		5
6A			X	X	X		X	6A
6B	X	X	X	X	X	X		6B
7F		X	X	X	X	X	X	7F
8					X	X	X	8
9N						X	X	9N
9V	X	X	X	X	X	X		9V
10A					X	X	X	10A
11A					X	X	X	11A
12F					X	X	X	12F
14	X	X	X	X	X	X		14
15B					X	X	X	15B
17F						X	X	17F
18C	X	X	X	X	X	X		18C
19A			X	X	X	X	X	19A
19F	X	X	X	X	X	X		19F
20						X	X	20
22F				X	X	X	X	22F
23F	X	X	X	X	X	X		23F
33F				X	X	X	X	33F
15A							X	15A
15C							X	15C
16F							X	16F
23A							X	23A
23B							X	23B
24F							X	24F
31							X	31
35B							X	35B

In green: serotypes inluded only in that vaccine
